# Examining community mental health providers' delivery of structured weight loss intervention to youth with serious emotional disturbance: An application of the theory of planned behaviour

**DOI:** 10.1111/hex.13357

**Published:** 2021-09-28

**Authors:** Thomas L. Wykes, Andrea S. Worth, Kathryn A. Richardson, Tonja Woods, Morgan Longstreth, Christine L. McKibbin

**Affiliations:** ^1^ Department of Psychology University of Wyoming Laramie Wyoming USA; ^2^ Present address: Thomas L. Wykes, Veterans Affairs Cheyenne Healthcare System 2360 E. Pershing Blvd Cheyenne WY 82001, USA

**Keywords:** overweight and obesity, serious emotional disturbance, theory of planned behaviour, weight loss interventions, youth

## Abstract

**Background:**

Rates of overweight and obesity are disproportionately high among youth with serious emotional disturbance (SED). Little is known about community mental health providers' delivery of weight loss interventions to this vulnerable population.

**Objective:**

This study examined attitudinal predictors of their providers' intentions to deliver weight loss interventions to youth with SED using the theory of planned behaviour.

**Design:**

This study used a cross‐sectional, single‐time‐point design to examine the relationship of the theory of planned behaviour constructs with behavioural intention.

**Setting and Participants:**

Community mental health providers (*n* = 101) serving youth with SED in the United States completed online clinical practice and theory of planned behaviour surveys.

**Main Variables Studied:**

We examined the relationship of direct attitude constructs (i.e., attitude towards the behaviour, social norms and perceived behavioural control), role beliefs and moral norms with behavioural intention. Analyses included a confirmatory factor analysis and two‐step linear regression.

**Results:**

The structure of the model and the reliability of the questionnaire were supported. Direct attitude constructs, role beliefs and moral norms predicted behavioural intention to deliver weight loss interventions.

**Discussion:**

While there is debate about the usefulness of the theory of planned behaviour, our results showed that traditional and newer attitudinal constructs appear to influence provider intentions to deliver weight loss interventions to youth with SED. Findings suggest preliminary strategies to increase provider intentions.

**Public Contribution:**

This study was designed and the results were interpreted as part of a larger, community‐based participatory research effort that included input from youth, families, providers, administrators and researchers. Collaborative discussions with community mental health providers and administrators particularly contributed to the study question asked as well as interpretation of results.

## 1 INTRODUCTION

Overweight and obesity (OW/OB) among youth are major global public health problems.^1^, ^2^ In the United States, the National Survey of Children's Health in 2016–2017 calculated the prevalence of OW/OB among randomly sampled children aged 10–17 years in the United States and found that 9.5 million of these youth were either overweight (15.2%) or obese (15.8%).^3^ A recent evidence report and systematic review of obesity screening for the U.S. Preventive Services Task Force also indicated that the prevalence of obesity among youth has increased over the past three decades. While the authors suggest that the rate of obesity may be stabilizing overall, they emphasized the importance of addressing OW/OB in youth as a public health priority.^4^

Work over the last two decades suggests that OW/OB may disproportionately affect youth with psychiatric disorders,^5^, ^6^, ^7^, ^8^ referred to by the Substance Abuse and Mental Health Services Administration (SAMHSA) as serious emotional disturbance (SED). For example, a recent large study utilizing the 2016 National Survey of Children's Health was conducted to examine the prevalence of overweight among youth aged 10–17 years across 19 chronic conditions (*n* = 10,997) compared to those without chronic conditions (*n *= 13,408). They found a significantly greater prevalence of overweight among youth with depression (40.7%), behaviour problems (39.3%) and anxiety (36.6%) relative to youth without these chronic conditions (27.8%).^9^ The authors of a smaller cross‐sectional chart review study of adolescents (*n = *114) admitted to a behavioural health partial hospitalisation programme found rates of overweight (25.4%) and obesity (30.0%) that were significantly higher than those of samples of youth in the general population of both the surrounding county and across the nation.^10^ In another study of youth aged 8–11 years, Lumeng et al.^11^, ^12^ found that clinically meaningful behaviour problems were independently associated with an increased risk of concurrent overweight and increased risk of becoming overweight among previously normal‐weight children.

### 1.1 Addressing OW/OB among youth with SED

Interventions are needed to address OW/OB among youth with both SED and OW/OB. Despite risk for long‐term deleterious outcomes associated with SED, and the need for specialized interventions for this vulnerable population, few programmes have been developed. A small body of research to address healthy lifestyle has shown promising health outcomes among emerging adult and adult populations with first‐episode psychosis and across both community and mental health centre settings.^13^, ^14^ Mental health providers have either led or collaborated in the delivery of these interventions. However, information about the involvement of key stakeholders (e.g., youth and families, mental health providers and administrators) in the development of these interventions is less clear. Mental health providers may also be uniquely positioned to contribute, along with researchers and both youth and families, to the development of an intervention designed to be implemented within existing mental health service systems. It is well known that mental health providers have knowledge and expertize in working with youth with SED and their family members, knowledge of the important system‐level influences and barriers to service delivery, knowledge of social determinants of health‐influencing outcomes in these populations and expertize in the self‐management and behaviour change strategies that are commonly used in mental health interventions.^15^, ^16^, ^17^, ^18^, ^19^ In general, however, the degree to which these professional stakeholders are ready and willing to engage vulnerable populations such as youth with SED and OW/OB and family members is less well known.

Ashby et al.^20^ examined provider readiness to address healthy lifestyles among 259 nonphysician, Australian, healthcare professionals. A total of 21 of these providers were psychologists and were serving adult mental health clients with OW/OB. The psychologists in the sample observed substantial deficits in perceived abilities to provide healthy lifestyle advice to clients, as well as low knowledge about weight loss, low confidence for setting weight loss goals and low confidence in making dietary and physical activity recommendations. Despite these doubts, 42% (*n = *8) of the psychologists in the sample reported providing dietary advice and 60% (*n* = 12) believed that doing so was within their professional role. Ashby et al.^20^ attributed providers' decisions to convey weight‐related healthy lifestyle advice to patients with OW/OB to the influence of several factors, including providers’ beliefs regarding the scope of their practice, their confidence in providing weight‐related healthy lifestyle advice and access to supportive resources. Although the study carried out by Ashby et al.^20^ is one of the first to examine engagement in and attitudes towards providing weight‐related lifestyle advice among mental health providers, their report of only descriptive data and unclear operationalization of the theory of planned behaviour constructs limited the inferential power of their results. In addition, the degree to which providers and the community of individuals with mental health disorders was involved in developing the survey questions was less clear.

### 1.2 The theory of planned behaviour: Understanding provider intentions

The theory of planned behaviour^21^ may be a valuable framework for understanding provider intentions to engage youth with SED and OW/OB and their families in weight loss interventions. While the theory of planned behaviour has received some criticism (e.g., limited validity, lack of ability to empirically disprove the theory, lacking sufficient belief altering guidelines)^22^ and other motivational theories have been put forward as alternatives (e.g., Health Action Process Approach),^23^ this particular theory has been widely used in previous research to efficiently characterize the decision‐making process regarding specific behaviours and to predict future decisions to perform those behaviours. Unlike other motivational theories, the Theory of Planned Behaviour has also been extended to studies of provider behaviour. A systematic review of 78 studies seeking to predict healthcare professionals' intentions to perform specific behaviours found that the theory of planned behaviour (or its parent theory, the theory of reasoned action) was the most commonly used model in investigations of healthcare professionals' intentions. The theory of planned behaviour also demonstrated the strongest association between theoretical components and the actual behaviours of providers.^24^ The theory of planned behaviour is founded on the assumption that individuals develop intentions to perform a target behaviour (i.e., behavioural intentions) that lead to engagement in the behaviour.^21^ Several psychological constructs contribute to the development of behavioural intentions. The theory states that salient beliefs drive the cognitive constructs that contribute to behavioural intentions. Salient beliefs include specific beliefs about (1) the target behaviour (i.e., behavioural beliefs), (2) others who would approve or disapprove of engaging in the behaviour (i.e., normative beliefs) and (3) the ability to control aspects of the behaviour (i.e., control beliefs). These salient beliefs correspond directly to the following cognitive constructs (i.e., direct attitude variables): (1) attitude towards the behaviour, (2) subjective norm and (3) perceived behavioural control. Attitude towards the behaviour refers to favourable or unfavourable appraisals held by an individual about the specific behaviour. Subjective norm refers to social pressure regarding whether or not to engage in the behaviour. This social pressure is influenced by the opinions of others whom the individual deems important. Finally, perceived behavioural control refers to an individual's appraisal of and corresponding beliefs about his or her own ability to carry out the behaviour in question.^21^, ^25^

The theory of planned behaviour also allows for the inclusion of additional constructs when there is sufficient evidence to support doing so. For example, the additional influence of role beliefs and moral norms on the behavioural intentions of healthcare providers has received some empirical support.^24^ These additional constructs stem from Triandis'^26^ theory of interpersonal behaviour. Role beliefs are defined as ‘… behaviors appropriate for persons holding a particular position in a group, society, or social system’,^26^ (p. 208) and moral norms are defined as ‘… feelings of personal responsibility regarding the performance… of a given action’^26^ (p. 94). In their review of healthcare provider behaviour, Godin et al.^24^ reported that role beliefs were a significant predictor of intention in 8 of 14 studies that used the construct. Moral norms were a significant predictor of intention in 10 of 14 studies that used the construct. The authors identified role beliefs and moral norms as among ‘the most consistently significant cognitive factors’ (p. 5) related to intention in the context of healthcare provider behaviour. More recent studies have also shown the value of moral norms in predicting intention to receive an human papillomavirus vaccine,^27^ to comply with hand hygiene^28^ and participate in regular leisure‐time physical activity among individuals with diabetes,^29^ among other behaviours.^30^

### 1.3 Aim of the present study

The present study was conducted by researchers in collaboration with a group of key stakeholders including youth and families, mental health providers, community mental health administrators and academic researchers. This study is one of several steps towards the development of a specialized intervention to promote healthy lifestyles among youth with SED and OW/OB. For this study, the group sought to characterize community mental health providers' engagement of youth with both SED and OW/OB and their family members in weight loss programmes as well as identify the key attitudinal predictors of providers' intentions to engage this vulnerable population in structured weight loss interventions. Understanding the attitudinal factors that may influence the availability of much‐needed and specialized health promotion services for youth with OW/OB and their family members is expected to provide additional avenues for provider education and programme development. We first hypothesized that each direct attitude construct (i.e., attitude towards the behaviour, subjective norm, perceived behavioural control) as well as added constructs (i.e., role beliefs and moral norms) would be positively associated with the intention to provide structured weight loss interventions to youth with SED and OW/OB. We then hypothesized that the intention to provide structured weight loss interventions to youth with SED and OW/OB would be positively associated with self‐reported history of providing such interventions. Given these specific aims and existing gaps in the literature, a measure was developed for use in the present study. As a result, additional aims of the present study included assessing and reporting the fit of the observed provider data to the expected factor structure.

## 2 METHODS

### 2.1 Sample

Community mental health providers who serve vulnerable youth with SED were recruited from eligible mental health centres in the United States. SED is defined by the United States SAMHSA as any youth from birth to age 18 who has a diagnosable mental, behavioural or emotional disorder that substantially interferes with or limits the youth's role or functioning in family, school or community activities.^31^ Eligible mental health centres were those that (1) provide mental health treatment services to children, adolescents, young adults or adults; (2) provide crisis or emergency treatment options; (3) operate in an outpatient setting; (4) provide specialty services for SED; and (5) provide internet‐based contact options for administration of study materials. Individuals who were 18 years of age or older, who worked as a mental health provider, who worked in an eligible mental health centre and who expressed informed consent were eligible to participate.

### 2.2 Measures

#### 2.2.1 Sociodemographics

A sociodemographic form was used to collect the personal and professional characteristics of all participants (e.g., age, occupation and years in practice).

#### 2.2.2 Theory of planned behaviour questionnaire

A 41‐item theory of planned behaviour questionnaire was developed for the study, based on published theory of planned behaviour guidelines,^25^, ^32^ and was revised by three experts in the field. The questionnaire addresses salient beliefs (i.e., behavioural beliefs, normative beliefs and control beliefs), direct attitude variables (i.e., attitude towards the behaviour, subjective norm and perceived behavioural control), role beliefs, moral norms and behavioural intention. Role beliefs and moral norms were added to the measure based on feedback from researchers with expertize in the theory. The salient belief items were identified in a previous elicitation study from this study group^33^ and were added to questions addressing the direct attitude and behavioural intention constructs of the theory of planned behaviour. A single item (i.e., “I provide structured weight loss intervention to my youth clients with SED and OW/OB”) measured engagement in the target behaviour. All items were structured as 5‐point, Likert‐type items, and were coded such that higher scores reflect more favourable beliefs and engagement in the target behaviour. For each scale, a summary score was calculated as the simple mean of the items.

#### 2.2.3 Clinical practice survey

A 26‐item survey, based partly on the measure used by Ashby et al.,^20^ collected information about engagement in weight‐related treatment activities (e.g., providers' assessment of weight and lifestyle behaviours, types of dietary and physical activity services provided). The survey included Likert‐type items (e.g., ‘For your youth clients with SED and OW/OB, how often do you directly address your client's weight in your sessions?’) and open‐ended questions (e.g., ‘What percentage of your youth clients with SED have OW/OB?’). The survey allowed for the calculation of frequency counts of reported weight‐related treatment activities and qualitative description of additional needs and preferences in relation to these behaviours.

### 2.3 Procedure

This study was conducted as part of a larger community‐based participatory research effort to develop a healthy lifestyle intervention for youth with SED and OW/OB and their family members. The tool that was used, intervention mapping (IM),^34^ is a community‐based, participatory model, including patient and public involvement, which serves as a blueprint for designing, implementing and evaluating an intervention based on theoretical, empirical and practical information. A key component of the IM protocol is the engagement of stakeholders in all phases of intervention development from identification of the problem, to planning for research and needs assessments, to identification of essential programme elements, to evaluation of the intervention. In this case, a stakeholder board comprising parents and youth (*n* = 4), community mental health providers (*n* = 4), administrators (*n* = 2) and researchers (*n* = 6) met on a monthly basis. Feedback on design and results from community mental health providers and administrators was sought and incorporated into this study.

Participants in this study were recruited from community mental health agencies listed in the United States SAMHSA national directory of mental health treatment facilities. The inclusion criteria were applied to all 50 states and yielded a list of 1989 entries. Sites were manually evaluated to verify eligibility for participation. Potential participants were contacted via email and/or website‐based contact forms.

All measures were administered through an internet‐based survey platform (i.e., Qualtrics).^35^ Prospective participants first navigated to a screening page to assess their inclusion criteria. All participants had the opportunity to indicate informed consent and to participate in the survey, which allowed administrators to review the survey even if they were not direct service providers. However, those who did not consent to participate were not included. The survey took an average of 20 min to complete. Responses for all survey questions other than identity and survey completion status were deidentified. Participants who completed the survey were entered in a raffle for one of 15 Amazon gift cards, each worth $20. The University of Wyoming Institutional Review Board approved this study. The study conforms to recognized standards of the US Federal Policy for the Protection of Human Subjects.

### 2.4 Data analysis

Descriptive statistics were calculated for all questionnaire items. Responses to the Clinical Practice Survey were dichotomized as ‘Never or Almost Never’ and ‘Rarely’ versus ‘Sometimes’, ‘Frequently’ and ‘Always or Almost Always’. All relevant variables were checked for normality (Kolmogorov–Smirnov test); transformations of nonnormal variables did not result in improvements in normality, so all analyses were performed with untransformed variables. Analyses were performed using SPSS version 23 and Mplus version 7.2.

#### 2.4.1 Theory of planned behaviour questionnaire psychometrics

The internal consistency reliability of the direct attitude, role beliefs, moral norms and behavioural intention scales was evaluated using Cronbach's *α*. Item–total correlations were also calculated. Pearson correlations were calculated between each item on each salient belief scale and the total score on its corresponding direct attitude scale to determine which beliefs have the strongest relationships with attitudinal constructs.^32^ Finally, construct validity for the direct attitude scales was tested with a confirmatory factor analysis and a maximum likelihood estimation approach. Model fit was evaluated with three tests:^36^ (1) standardized root mean square residual (SRMSR), (2) root mean square error of approximation (RMSEA) and (3) the comparative fit index (CFI).

#### 2.4.2 Direct attitude constructs as predictors of behavioural intention

A two‐step linear regression was conducted to evaluate the prediction of behavioural intention by direct attitude constructs. The three direct attitude scales (i.e., attitude towards the behaviour, subjective norm and perceived behavioural control) were entered in Block 1, and the role beliefs and moral norms scales were entered in Block 2. The *R* ^2^ change statistic was calculated to evaluate the incremental change in the overall model caused by adding these constructs. A Pearson correlation was also computed between behavioural intention and engagement in the behaviour. For all analyses, alpha was set to *p* < .05, and all results were two‐tailed.

## 3 RESULTS

### 3.1 Sample

A total of 578 (59.3%) sites fulfilled the inclusion criteria. Participants were distributed across at least 49 unique sites (missing *n* = 3). Participants (*n *= 101) were located across 25 states, with the largest representation in New Hampshire (*n* = 10) and Washington (*n* = 10) states. The majority were female, had obtained a master's degree and were employed as a licensed professional counsellor (see Table 1).

**Table 1:** Sample characteristics (*n* = 101)

Characteristic	*M* (SD)	*n* (%)
Age^a^	37.6 (10.2)	
Years in practice	9.3 (8.7)	
Female gender		87 (86.1)
Primary role^b^		
Licensed professional counsellor		39 (38.6)
Social worker		23 (22.8)
Marriage and family therapist		9 (8.9)
Psychiatrist		6 (5.9)
Psychologist		6 (5.9)
Nurse		2 (2.1)
Other clinician		12 (12.4)
Education level^c^		
Doctoral		13 (12.9)
Master's		50 (49.5)
Bachelor's		10 (9.9)
Other^d^		25 (24.8)

^a^ *n* = 96.

^b^ *n* = 97.

^c^ *n* = 98.

^d^ Twenty‐five participants noted some aspect of their occupation or licensure rather than a degree level.

### 3.2 Clinical practice and needs

Nearly one‐half of the providers (*n *= 47, 47%) reported directly addressing weight with clients in some capacity; 44% (*n *= 44) reported dispensing specific dietary advice; and 70% (*n* = 70) dispensed specific physical activity advice. A majority of the sample (*n *= 86, 86%) reported addressing psychosocial issues related to their clients' weight (e.g., bullying). However, nearly all participants (*n *= 91, 91%) reported that they ‘Never’ use a manualized weight loss intervention. Providers reported strongest preferences for (*n *= 50, 50%) and highest use of (*n *= 59, 59%) the internet as a source for obtaining information about OW/OB and its treatment. Frequently reported barriers to receiving training included few opportunities for training on this topic (*n *= 44, 44%) and little knowledge of how to access such training (*n *= 44, 44%). A large majority of providers reported that their workplace has neither guidelines pertaining to providing weight loss interventions (*n *= 92, 92%) nor a system for referring clients for weight loss treatment (*n *= 69, 69%).

### 3.3 Theory of planned behaviour questionnaire scores and scale reliability

Reliability for the direct attitude scales varied. Reliability was acceptable for attitude towards the behaviour (*α* = .84) and subjective norm (*α* = .72), but poor for perceived behavioural control (*α* = .53). Removing two items with poor fit (i.e., ‘The decision for me to provide structured weight loss intervention to my youth clients with SED and OW/OB is beyond my control’; ‘Whether I provide structured weight loss intervention to my youth clients with SED and OW/OB is entirely up to me’) from the perceived behavioural control scale resulted in stronger internal consistency reliability (*α* = .80) and corrected item–total correlations (ranging from *r* = .57 to *r* = .70). Role beliefs had acceptable reliability (*α* = .77), while moral norms had poor reliability (*α* = .57). Removing one item with poor fit (i.e., ‘I do feel/would feel guilty about providing structured weight loss intervention to my youth clients with SED and OW/OB’) from the moral norms scale resulted in stronger internal consistency reliability (*α* = .72) and correlation between the two remaining items (*r* = .57). Finally, the behavioural intention scale demonstrated strong internal consistency reliability (*α* = .87).

### 3.4 Theory of planned behaviour questionnaire scale validity/factor structure

Confirmatory factor analysis was conducted with items measuring attitude towards the behaviour, subjective norm and perceived behavioural control (after removing two items with poor fit). Results indicated adequate‐to‐good model fit^37^, ^38^ across the three goodness‐of‐fit indices. The absolute model fit result (SRMSR = 0.067), the parsimony correction analysis result (RMSEA = 0.073) and the comparative fit analysis (CFI = 0.949) all fell within ranges indicative of good fit. Thus, the confirmatory factor analysis supported the factor structure of the theory of planned behaviour model as applied in the present study.

### 3.5 Direct attitude scales as predictors of behavioural intention

Descriptive statistics were calculated for each scale. Means and standard deviations revealed moderate to high endorsement of items on attitude towards the behaviour (*M* = 3.98, *SD = *0.70), perceived behavioural control (*M* = 3.01, *SD* = 0.89), moral norm (*M* = 3.18, *SD* = 0.84) and role beliefs (*M* = 2.55, *SD* = 0.81) scales and lower endorsement of items on the subjective norm (*M* = 1.79, *SD *= 0.71) scale. Means and standard deviations were also calculated for behavioural intention to provide structured weight loss interventions (*M* = 2.61, *SD* = 0.93) and engagement in this behaviour (*M* = 1.61, *SD* = 0.95).

The associations between the direct attitude scales and behavioural intention were evaluated using a two‐step linear regression analysis (see Table 2). The direct attitude scales (i.e., attitude towards the behaviour, subjective norm and perceived behavioural control) were entered into Block 1 simultaneously. The model accounted for approximately 48% of the variance, and each direct attitude scale predicted behavioural intention. Role beliefs and moral norm scales were entered into Block 2, resulting in approximately 67% of the variance being accounted for by the model, a statistically significant increase. In Block 2, all predictors except attitude towards the behaviour were statistically significant. Finally, a Pearson correlation was computed between behavioural intention and engagement in the behaviour. The variables were significantly correlated (*r* = .63, *p* < .01).

**Table 2:** Predictors of intention to provide a structured weight loss intervention (*n* = 99)^a^

Model	*β*	*R* ^2^	Δ *R* ^2^	*p*
Block 1		.542	.542	<.001
Attitude towards the behaviour	.338			<.001
Subjective norm	.288			.001
Perceived behavioural control	.381			<.001
Block 2		.693	.151	<.001
Attitude towards the behaviour	.127			.082
Subjective norm	.155			.033
Perceived behavioural control	.234			.001
Role belief	.217			.011
Moral norms	.372			<.001

^a^ Sample size was *n *= 99 for the regression analysis, due to missing data.

## 4 DISCUSSION

The primary goal of this study was to examine the relationship between community mental health providers' attitudes and their intentions to engage in the delivery of structured weight loss interventions designed for vulnerable youth with SED and OW/OB. This study examined the value of attitudinal constructs, outlined by the theory of planned behaviour, to understand provider intentions. It is important to note that there has been debate in the literature regarding the ongoing usefulness of the theory of planned behaviour in general. Specifically, a paper by Sneihotta et al.^22^ called into question the validity of the theory of planned behaviour and noted that its parsimony may limit its ability to explain a sufficient proportion of variance in behaviour. We used a systematic approach^25^ to design a measure of the theory of planned behaviour to measure attitudinal influences of community mental health providers on their intentions to engage youth with SED and OW/OB in structured weight loss interventions. Results of a confirmatory factor analysis and two‐step linear regression in the present study indicated that the measure was consistent with the theory of planned behaviour and showed support for the relationship between theory‐driven attitudinal constructs and community mental health providers' behavioural intentions. Results from this study were consistent with previous literature examining provider behavioural intentions.^24^

The efforts of researchers to expand the theory of planned behaviour to include new and relevant constructs and criticisms of others identify the lack of ability to falsify both the traditional as well as newly added constructs. Ajzen^21^ himself acknowledged flaws of the theory, and indicated that a determinant should not be introduced unless it offers more variance than the others already included in the model. In our study, expert feedback was solicited regarding the theory‐driven, attitudinal measures, which resulted in the addition of two new attitudinal constructs to the model (i.e., role belief and moral norm). The inclusion of these constructs significantly added to the prediction behavioural intention even above the substantial variability accounted for by the traditional model. This finding was also consistent with previous research.^39^ However, in the present study, the addition of the moral norms construct to the regression model also resulted in attitude towards the behaviour becoming a weaker and nonsignificant predictor of behavioural intention. Multicollinearity was not a factor in this change. It is possible that some of the traditional constructs may hold less value when compared to newer constructs such as moral norms. It is also possible that the role of individual constructs may vary by target behaviour.

In addition to attitudinal constructs, a survey of existing practices of community mental health providers in this study provided information about what is currently happening in practice regarding OW/OB among clients with mental health disorders. The survey showed that providers in this study directly addressed weight concerns with clients and most often did so by dispensing dietary or physical activity suggestions or advice. Many providers also reported a lack of training opportunities to enhance their knowledge and skills for addressing weight concerns or creating health behaviour change. These results are somewhat different from a study conducted in an adult sample from Australia^20^ in which a minority of providers reported dispensing weight‐related advice. While unclear, sources of discrepancy between our results and those of Ashby et al.^20^ may include important differences in methodology, sampling procedures (including the countries in which the studies were conducted) and target treatment considered. The results of our study indicate that workforce skill development may be needed to enhance providers' abilities to increase access to evidence‐based, structured weight management services designed for youth with SED and OW/OB.^40^

### 4.1 Limitations and future research directions

The present study is one of the first, to our knowledge, to design and implement a theory of planned behaviour‐informed measure of provider intentions to provide structured weight loss interventions to youth with SED and OW/OB and do so within the context of a community‐based participatory approach to intervention development. While the project hypotheses were supported, the results should be considered within the context of their limitations. First, behavioural intention in the present study focused on the broad concept of providing weight loss intervention in general. There may be important differences in providers' intentions to address diet and to address physical activity. Future studies should address weight‐related interventions (e.g., diet and physical activity) separately to reduce ambiguity and to elucidate causes for differential engagement in various aspects of weight‐related treatments. Second, limitations related to recruitment procedures and sample characteristics should be noted. First, recruitment was conducted via impersonal and electronic means. It is possible that centres and individuals who chose to participate were more likely than nonparticipants to engage in weight management discussions with their clients. Lack of data from nonparticipants limits our understanding of the generalisability of the findings. In addition, participants were also engaged in the provision of mental health services within the community mental health system in the United States and may not reflect attitudinal influences on provider behaviours in other mental health systems in other countries. The present study was also limited by the lack of an objective measure of clinician engagement in the target behaviour. This construct was addressed with a single self‐report item scored on a 5‐point, Likert‐type scale. A single, self‐report item was not deemed sufficient to support more complex analyses such as path modelling or structural equation modelling. Future studies could include a more thorough measures of behavioural engagement. It would be ideal if such an indicator could include an outcome based on objective evidence, such as activity logs of direct researcher observation. Finally, the identification of cross‐sectional predictors of behavioural intention provides only promising ideas and generates future hypotheses for examination in future studies. Longitudinal studies and experimental studies may identify predictors of behavioural intention over time. Experimental examination of strategies to alter provider attitudes regarding engagement in structured weight management services may also add to the theory's utility for use in practice in community mental health settings.

### 4.2 Summary and conclusions

In summary, the present study, included within a larger community‐based, participatory intervention development effort, examined attitudinal predictors of community mental health providers' intentions to engage in weight management interventions with youth clients with SED and OW/OB. Youth with SED who experience OW/OB comprise a vulnerable population that lacks access to specialty interventions to meet their specific needs. This population may be especially likely to benefit from the integration of weight loss treatment into the mental health setting, as they may have no other regular access to these services. The results of the present study suggest that peers with whom providers interact as well as providers' perceived control in offering structured weight loss interventions are potentially important factors in their decisions to offer these services. Importantly, the additional value of constructs such as role belief and moral norms suggests that providers who are reluctant to offer these services may have concerns about whether addressing bodyweight is within their role and whether it is the right thing to do. Our previous qualitative work with youth, parents and providers^41^ echoes these results. Specifically, interviews with providers revealed concerns about whether they should address OW/OB amid mental health concerns as well as fear that they may offend their clients when discussing bodyweight. Results from these studies suggest that providers may benefit from workforce education regarding how to effectively discuss and monitor OW/OB among their clients. Indeed, the results from our study indicated that many providers may already be discussing healthy lifestyles and offering advice. Further formative and experimental research, collaborating with youth and families as well as providers, may help to develop workforce messaging and specific training to increase their capacity and willingness to integrate evidence‐based strategies to reduce OW/OB among youth with SED as a part of the overall plan of care.

## CONFLICT OF INTERESTS

The authors declare that there are no conflict of interests.

## AUTHOR CONTRIBUTIONS

Thomas Wykes was responsible for conceptualisation of the study and design, data collection, analysis and initial manuscript writing. Andrea S. Worth was responsible for substantial manuscript conceptualisation, writing and revision. Kathryn A. Richardson was responsible for literature review and substantial contribution to writing and revision of the introduction. Tonja Woods was responsible for substantive contribution to interpretation of results and writing of the discussion. Morgan Longstreth was responsible for literature review and manuscript editing. Christine L. McKibbin assisted with study conceptualisation, study design, oversight of data collection, analysis, interpretation, manuscript writing and editing.

## Data Availability

The data that support the findings of this study are available on request from the corresponding author. The data are not publicly available due to privacy or ethical restrictions.
